# Dental magnetic resonance imaging for bone loss assessment and disease activity classification in severe periodontitis

**DOI:** 10.1186/s13244-025-02004-7

**Published:** 2025-06-26

**Authors:** Arne Lauer, Luisa Schulte, Artid Skenderi, Nouha Tekiki, Alexander Juerchott, Meysam Sohani, Maurice Ruetters, Franz Sebastian Schwindling, Peter Rammelsberg, Mathias Nittka, Sabine Heiland, Martin Bendszus, Tim Hilgenfeld

**Affiliations:** 1 Department of Neuroradiology, Heidelberg University Hospital, Heidelberg, Germany; 2 Private Practice, Schwalmtal, Germany; 3 Section of Periodontology, Department of Operative Dentistry, Heidelberg University Hospital, Heidelberg, Germany; 4 Department for Prosthetic Dentistry, Medical University Innsbruck, Innsbruck, Austria; 5 Department of Prosthodontics, Heidelberg University Hospital, Heidelberg, Germany; 6 Magnetic Resonance, Siemens Healthineers AG, Erlangen, Germany

**Keywords:** Magnetic resonance imaging, Periodontal disease, Periodontitis

## Abstract

**Objectives:**

To evaluate the reliability and accuracy of dental MRI (dMRI) for volumetric infrabony and furcation bone loss compared to cone-beam computed tomography (CBCT) and to correlate to clinical signs of inflammation in patients with severe periodontitis.

**Methods:**

In this cross-sectional study nineteen patients with severe periodontitis underwent standardized clinical examination as well as pre-treatment CBCT and 3T-dMRI. Bone lesion volumetry was performed in CBCT, contrast-enhanced-T1-weighting (T1W + C) and T2-weighting (T2W) dMRI. Lesions whose T2W signal significantly exceeded T1W/CBCT margins (indicating excessive edema) were classified as T2W-mismatch. Volumetric data were compared to clinical findings.

**Results:**

Ten female and nine male patients with 253 bony lesions were examined. Reliability for bone lesions was highest in CBCT (ICC [95% CI] T1W + C/T2W/CBCT: 0.78 [0.74–0.83]/0.82 [0.77–0.85]/0.87 [0.94–0.89]). Overall, T1W + C and T2W dMRI strongly correlated with CBCT (r_s_ = 0.86 [95% CI: 0.82–0.89], *p* < 0.001 and r_s_ = 0.91 [95% CI: 0.88–0.93], *p* < 0.001 respectively) but volume was significantly overestimated by dMRI (median percentage error of T1W + C-T2W: 19–55%). A T2W-mismatch was found in 44.1% and correlated with bleeding (85.8% vs. 70.9%, *p* = 0.005), giving 47.5% sensitivity and 71.2% specificity.

**Conclusions:**

While dMRI offers good reliability, T2W- and to a lesser extent T1W + C imaging overestimate infrabony and interradicular periodontal bone lesion volumetry compared to CBCT. While this could increase the risk of overtreatment, dMRI detects periodontal inflammation beyond areas of bone loss, and T2W-mismatch is closely related but not identical to signs of active inflammation in clinical examination. This may provide additional diagnostic information and could serve as a supplemental tool for higher-risk patients.

**Critical relevance statement:**

Dental MRI excels in detecting inflammation beyond bone loss, identifying high-risk tissue. This study assesses reliability in evaluating periodontitis-related bone loss, highlighting its tendency to overestimate lesion volume. A novel “mismatch lesion pattern” was observed, potentially linked to disease activity.

**Key Points:**

- Dental MRI (dMRI) reliably assesses bone loss in periodontitis but overestimates volume vs. cone-beam computed tomography (CBCT).
- dMRI detects excess bone marrow edema, indicating inflammation beyond visible bone loss.
- dMRI could aid periodontal diagnosis and guide targeted therapeutic interventions.

**Graphical Abstract:**

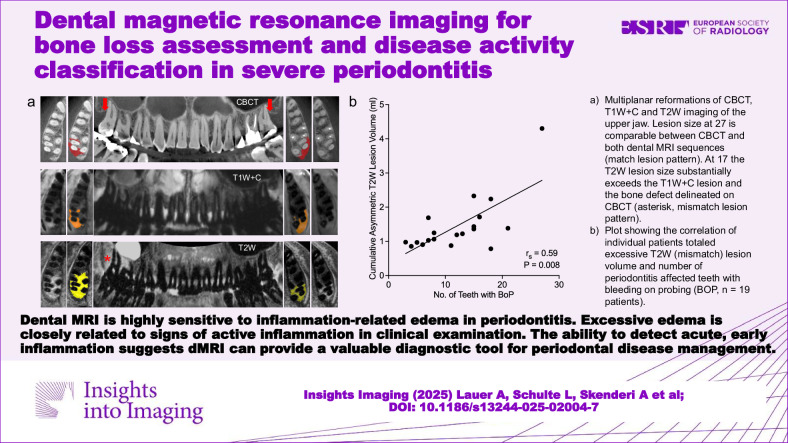

## Introduction

The loss of the teeth supporting alveolar bone due to chronic inflammation is a defining characteristic of severe periodontitis. This condition is estimated to affect approximately 11% of adults and significantly impacts the quality of life related to oral health [1–3]. Periodontitis is a complex disease involving bacterial pathogens and immune responses and is characterized by recurrent episodes of inflammation [4]. It locally affects the supporting tissues of the teeth, leading to a gradual breakdown of tooth attachment and the surrounding bone, but also leads to systemic inflammation [5].

While two-dimensional x-rays are common for assessing bone loss, they lack volumetric information and are limited by superimposition and distortion. Cone-beam computed tomography (CBCT) overcomes some of these limitations and is used to assess the extent of periodontal bone tissue loss [6, 7]. But it only reveals late stages of the disease where tissue degradation has already occurred [8]. X-ray-based examinations are very limited in providing insights into the degree of active inflammation, the viability, or the potential for salvage of the involved radio-lucent tissues of the alveolar bone [9]. Further, these examinations contribute significantly to patient radiation exposure [10].

Active inflammation in periodontal disease results in localized increases in tissue water content, characterized by hyperemia, interstitial edema, and infiltration of mononuclear cells [11]. MRI-based T2-weighted imaging (T2W) is highly sensitive to changes in regional water content, and contrast-enhanced T1-weighted imaging (T1W + C) indicates inflammation-related regional breakdown of the blood-tissue barrier of the microvasculature. In comparison to CBCT, T1W dental MRI (dMRI) with and without contrast enhancement has demonstrated excellent diagnostic agreement and reliability in evaluating furcation involvement and clinically assessed probing depths (PD) in patients with periodontitis [12, 13]. Additionally, dMRI can delineate treatment-responsive intraosseous edema extending beyond areas of bone loss in patients with generalized periodontitis or periapical lesions and has been shown to surpass the capabilities of 2D x-ray-based techniques [14–17]. Previous MRI studies in periodontitis compared the accuracy of linear bone loss measurements for T1-weighted sequences (with and without use of contrast agent) but did not assess three-dimensional bone lesion volumes [14, 15]. The value of a combined analysis of T2-weighted and contrast-enhanced T1-weighted imaging in direct comparison with CBCBT is so far unknown.

Accurately visualizing inflammatory infiltration and understanding its relationship to the loss of tooth-supporting tissues is crucial. This information can provide insights into the stage, the activity of the disease and its spatial extent. This could guide targeted treatment strategies and potentially serve as a biomarker for disease monitoring. To clinically assess the disease, attachment loss (CAL) and probing pocket depths (PD) are measured to determine its extent, while reactive bleeding on probing (BOP) serves as a clinical indicator of inflammatory activity [18]. BOP has low specificity and sensitivity as it typically does not distinguish gingivitis from periodontitis, and on the other hand, tissues may be inflamed yet non-bleeding [19]. As a potential strength, multimodal dMRI could provide both cumulative (extent of the bone loss) and momentary (extent of inflammation-related bony edema) diagnostic information at the same time. Thereby, dMRI could help differentiate between stable and unstable (active) lesions, addressing an ongoing diagnostic gap in established periodontitis diagnostic.

The objective of this study is to is to evaluate if dMRI detects acute inflammatory infiltration of the periodontal bone by (1) comparing periodontal lesion volumetrics derived from dMRI (T2-weighted imaging and contrast-enhanced T1-weighted imaging) with volumetric infrabony and interradicular bone loss quantified by CBCT and (2) by correlation of these findings with clinical assessments in patients with periodontal disease.

## Methods

### Ethics approval and consent to participate

The prospective study was approved by the local research ethics committee of the University of Heidelberg (S-452/2010). All research was performed in accordance with local guidelines and regulations and in accordance with the Declaration of Helsinki. Written informed consent was obtained from all patients.

### Patients and clinical examinations

This cross-sectional, single-center study included patients with severe periodontitis (generalized periodontitis stage III or higher) using high-resolution 3D T2W and T1W MRI data from a prospectively enrolling study between September 2017 and December 2022. Only patients untreated for periodontitis in the prior 3 months with an available CBCT were included. Exclusions included MRI contraindications, age under 18, pregnancy, claustrophobia, and incomplete or motion-degraded MRI data.

Two experienced dentists (M.S. and F.S.S.), calibrated before the study, conducted clinical exams. Probing pocket depths (PD) and clinical attachment levels (CAL) were measured to the nearest millimeter at six sites (mesiobuccal, buccal, distobuccal, distopalatal, palatal, mesiopalatal) using a periodontal probe (HS-Parodontometer Figur CP15; Henry Schein Inc.). Bleeding on probing (BOP) was recorded at each site, with BOP percentage calculated as the number of bleeding sites divided by the total sites probed. Periodontitis diagnosis followed the 2017 World Workshop criteria, where interproximal attachment loss of ≥ 1 mm at ≥ 2 teeth was required [18]. Teeth were rated as affected if CAL ≥ 1 mm and probing depths ≥ 4 mm were found at any site.

### CBCT examinations

CBCT images were acquired using a 3D Accuitomo 170 system (J Morita) with the following settings: Cylindrical volume range: 4 × 4 to 8 × 8 cm^2^; tube voltage: 90 kV; tube current: 5 mA; rotation: 360°; scanning time: 17 s; isotopic voxel size: 0.16 mm.

### Magnetic resonance imaging

Dental MRI was performed on the same day as CBCT. All MRI scans were conducted on a 3-Tesla MRI system (MAGNETOM Trio; Healthineers AG, Erlangen, Germany) using a dedicated 15-channel dental coil (Mandibula; Noras MRI products GmbH).

A 3D fat-saturated T2-weighted (T2W) multi-slab acquisition with view-angle tilting gradient, based on a sampling perfection with application-optimized contrasts using different flip-angle evolution (MSVAT-SPACE) prototype sequence (Sequence parameters: 0.6 mm isovolumetric voxel size; time of echo, TE: 236 ms; time of repetition, TR: 2500 ms; time of inversion, TI: 200 ms; field of view, FoV: 191 × 151 mm^2^; receiver bandwidth: 521 Hz/pixel; acquisition matrix: 320 × 252; slice thickness, ST: 0.6 mm; number of sections, NOS: 104 at 425 s acquisition time) and a single 3D T1-weighted (T1W + C) isotropic Volumetric Interpolated Breath-hold Examination (VIBE) sequence with Dixon-based fat suppression after intravenous administration of gadolinium (0.7 mm isovolumetric voxel size; TE: 2.45 ms; TR: 15.6 ms; flip angle, FA: 14.9°; receiver bandwidth: 601 Hz/pixel; FoV: 223 × 153 mm^2^; acquisition matrix: 220 × 320; ST: 0.7 mm; NOS: 80 at 362 s acquisition time) were performed.

### Image processing and lesion segmentation

**Fig. 1:**
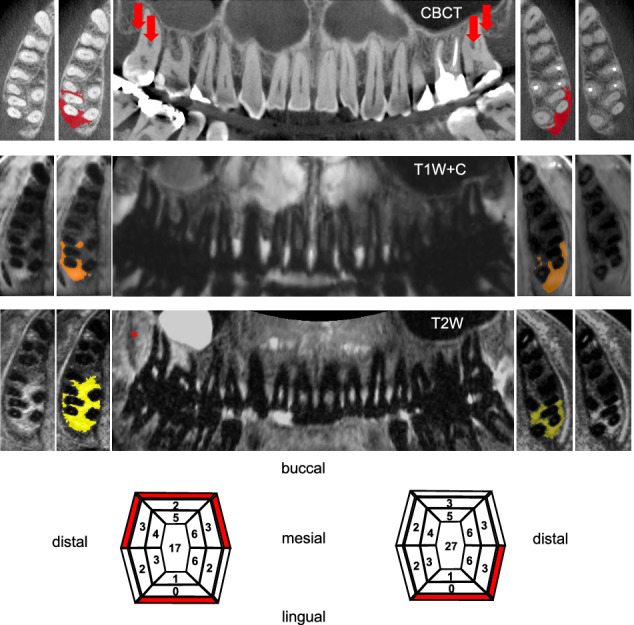
Match vs. mismatch lesion pattern based on T2W and T1W + C dMRI. Multiplanar reformations of CBCT, T1W + C and T2W imaging of the upper jaw of a female patient, 57 years of age, with severe periodontitis. The pictogram illustrates from inside to outside the probing depth, clinical attachment level and results of bleeding on probing (red = positive) for 17 and 27. There is obvious infrabony and subtle interradicular bone loss seen in the CBCT at 17 and 27(arrows). Lesion size at 27 is comparable between CBCT and both dMRI sequences (match lesion pattern). At 17, however, T2W lesion size substantially exceeds the T1W + C lesion and the bone defect delineated on CBCT (asterisk, mismatch lesion pattern). T2W and T1W + C dMRI, T2-weighted and contrast-enhanced T1-weighted, dental magnetic resonance imaging; CBCT, cone-beam computed tomography

All DICOM files were anonymized and converted to NIfTI format. T2W and T1W + C sequences were co-registered with CBCT using the 3D-SLICER landmark registration tool (https://www.slicer.org). Volumes of interest (VOIs) were placed using 3D-SLICER, following a two-step semi-automated protocol: initially, a threshold was set to exclude normal bone marrow voxels, and then the lesion was manually delineated (Fig. 1). Periodontal bone loss in CBCT was defined as infrabony and interradicular bone loss from the alveolar ridge if periodontal widening exceeded 0.2 mm. Lesions were detected on CBCT, with VOI placement for MRI restricted to planes showing these bone losses. Two independent readers (A.L. and L.S.), blinded to clinical data, performed VOI placement three times with at least a 4-week interval to prevent learning bias. The average lesion volume was used for statistical analysis.

### Mismatch lesions

Lesions were defined to show a mismatch pattern if the percentage increase of volumetrics delineated based on signal abnormalities on T2W maps exceeded the upper limit of the 95% CI of the T1W delineated lesions.

### Statistical analysis

Reliability was assessed and rated using intraclass correlation coefficients (ICCs) [20]. Agreement between readers and imaging methods was evaluated with Bland–Altman analysis [21]. The Shapiro–Wilk test assessed normality, and Levene’s test checked variance homogeneity. Spearman’s correlation assessed lesion volumes and PD correlations. A forced-entry linear regression analyzed whether T2W lesion excess, independently of smoking history, correlated with BOP or BOP%. Results were presented with coefficients (β), 95% confidence intervals (CI), and *p*-values. Friedmann and Kruskal–Wallis tests compared lesion volumes, and Fisher’s exact test evaluated frequencies of mismatched lesions and BOP at any one or more of the 6 probing sides. Two-tailed *p*-values < 0.05 were considered significant. Statistical analysis was performed using GraphPad Prism 10.0.2 and SPSS 22.

## Results

### Comparisons of imaging parameters dMRI vs. CBCT

A total of 19 patients were included in this study. Table 1 summarizes the patient and lesion characteristics of 285 periodontal lesions detected by clinical examination and/or CBCT. Clinical examination identified 253 teeth affected by periodontal attachment loss. CBCT reads showed infrabony and interradicular bone loss at 276 locations. CBCT was negative for bone loss in 9 (3.6%) of the clinically detected teeth and positive for bone loss in 32 (11.2%) teeth that were not classified as clinically affected. Thirteen (4.6%) of the affected sites could not be evaluated with dMRI due to metallic artifacts.

**Table 1:** Baseline characteristics of patients with severe periodontitis

Baseline characteristics of patients with severe periodontitis (*n* = 19)					
Age (median years, IQR)				53.38 (47.37–60.12)	
Female sex, *n* (%)				10 (53)	
Smoking history, *n* (%)				3 (16)	
Diabetes mellitus, *n* (%)				4 (21)	
Clinical periodontal lesions, median *n* (IQR)				11 (9–17)	
Clinical findings in teeth with periodontal lesions:					
Clinical attachment loss, median mm (IQR)				6 (5–8)	
Max. probing depth, median mm (IQR)				5 (4–6)	
Per patient number of teeth with probing depth > 5 mm, median *n* (IQR)				5 (2–7)	
Per patient percentage of bleeding probing sites, median % (IQR)				20.14 (10.12–28.67)	
Per patient number of teeth with bleeding on probing, median *n* (IQR)				10 (7–15)	
Imaging characteristics of teeth with periodontal lesions:					
Periodontal lesions per patient on CBCT, median *n* (IQR)				15 (12–18)	
CBCT lesion volume, median mL (IQR)				0.045 (0.022–0.092)	
T1W + C lesion volume, median mL (IQR)				0.062 (0.036–0.108)	
T2W lesion volume, median mL (IQR)				0.072 (0.041–0.131)	
Teeth by type:					
	*n* (%)	CBCT lesion median mL (IQR)	T1C lesion volume, median mL (IQR)		T2 lesion volume, median mL (IQR)
Incisor	63 (22)	0.023 (0.014–0.049)	0.042 (0.021–0.064)		0.043 (0.026–0.066)
Canine	35 (13)	0.030 (0.019–0.047)	0.042 (0.032–0.066)		0.051 (0.039–0.082)
Premolar	71 (25)	0.035 (0.019–0.067)	0.044 (0.030–0.082)		0.054 (0.035–0.101)
Molar	116 (40)	0.087 (0.047–0.149)	0.105 (0.065–0.172)		0.131 (0.077–0.201)

*CBCT* cone-beam computed tomography, *T1W* *+* *C* magnetic resonance fat-saturated contrast-enhanced T1-weighted imaging, *T2W* magnetic resonance fat-saturated T2-weighted imaging

Lesion volumetrics showed “good” agreement between readers for both modalities. Highest reliability was noted in CBCT (inter-rater ICC: 0.87 [95% CI: 0.89–0.94], intra-rater ICC: 0.91 [95% CI: 0.89–0.93]) and better in T2W vs. T1W + C (T2W inter-rater ICC: 0.82 [95% CI: 0.77–0.85], T2W intra-rater ICC: 0.90 [95% CI: 0.88–0.92] and T1W + C inter-rater ICC: 0.78 [95% CI: 0.74–0.83], T1W + C intra-rater ICC: 0.88 [95% CI: 0.85–0.91]). The range of agreement between readers and reads was most precise for CBCT (inter-rater lower to upper limit of agreement/bias: −0.051 to 0.081 mL/0.015 mL, intra-rater: −0.048 to 0.065 mL/0.008 mL), while T2W imaging showed less consistency between reads (inter-rater lower to upper limit of agreement/bias: −0.088 to 0.123 mL/0.017 mL, intra-rater: −0.063 to 0.085 mL/0.011 mL). The agreement range for T1W + C imaging fell between CBCT and T2-weighted imaging (inter-rater lower to upper limit of agreement/bias: −0.085 to 0.094 mL/0.005 mL, intra-rater: −0.057 to 0.069 mL/0.006 mL).

**Fig. 2:**
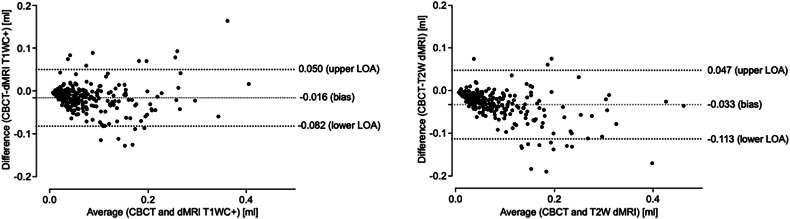
Comparison of CBCT and dental MRI lesion volumetrics. Bland–Altman plots of the differences between CBCT and T1W + C-dMRI (left) and T2W-dMRI (right) for delineated periodontal lesion volumes. The fine dotted lines represent the mean of all differences (bias) with negative values representing an dMRI-based overestimation of infrabony and interradicular bone loss compared to CBCT, and the coarse dashed lines the 95% LOA. CBCT, cone-beam computed tomography; dMRI, dental magnetic resonance imaging; lower LOA, lower limit of agreement; upper LOA, upper limit of agreement

Overall, a strong positive correlation of CBCT and dMRI-based lesion volumetrics was observed (T1W + C vs. CBCT: r_s_ = 0.86 [95% CI: 0.82–0.89], two-tailed *p* < 0.001 and T2W vs. CBCT: r_s_ = 0.91 [95% CI: 0.88–0.93], two-tailed *p* < 0.001, Spearman’s correlations). In direct comparison, however, dMRI lesion volumes were significantly larger compared to CBCT (median mL [IQR] for CBCT: 0.045 [0.022–0.092] vs. T1W + C: 0.062 [0.036–0.108] vs. T2W: 0.072 [0.041–0.131], Friedman test between group differences *p* < 0.001, Dunn’s multiple comparisons test CBCT vs. T1W + C adjusted *p* < 0.0001 and CBCT vs. T2W adjusted *p* < 0.001, respectively), with a mean absolute overestimation (bias) of 0.016/0.033 mL or median percentage error of 37 vs. 55% for the T1W + C/T2 sequence (Fig. 2).

A large fraction of all lesions 116/263 (44.1%) showed T2W signal abnormalities extending beyond the 95% CI of delineated CBCT bone loss volumes (lesion excess of > 64%). By the same definition, we found larger T2W lesions as compared to T1W + C lesions in 114 locations [40.0%], respectively (further termed mismatch lesion pattern). At least one of these mismatch lesions was present in every patient (median *n*, [IQR]: 6 [4–10], median %, [IQR]: of individual patient lesions: 50 [33–69]). If only match lesions were compared to CBCT, the mean absolute overestimation (bias) is reduced to 0.008/0.022 mL or a median percentage error of 18.9 and 28.1 for the T1W + C and T2W sequence.

### Comparisons of dMRI findings to clinical parameters

The imaging-based lesion volumes were positively correlated with maximum PD (CBCT vs. PD: r_s_ = 0.49 [95% CI: 0.39–0.58], *p* < 0.001, T1W + C vs. PD: r_s_ = 0.52 [95% CI: 0.42–0.61], *p* < 0.001 and T2W vs. PD: r_s_ = 0.55 [95% CI: 0.46–0.63], *p* < 0.001, Spearman’s correlations). Correlations to CAL were slightly weaker (CBCT vs. CAL: r_s_ = 0.48 [95% CI: 0.38–0.57], *p* < 0.001, T1W + C vs. CAL: r_s_ = 0.50 [95% CI: 0.41–0.59], *p* < 0.001 and T2W vs. PD: r_s_ = 0.52 [95% CI: 0.42–0.60], *p* < 0.001, Spearman’s correlations).

**Fig. 3:**
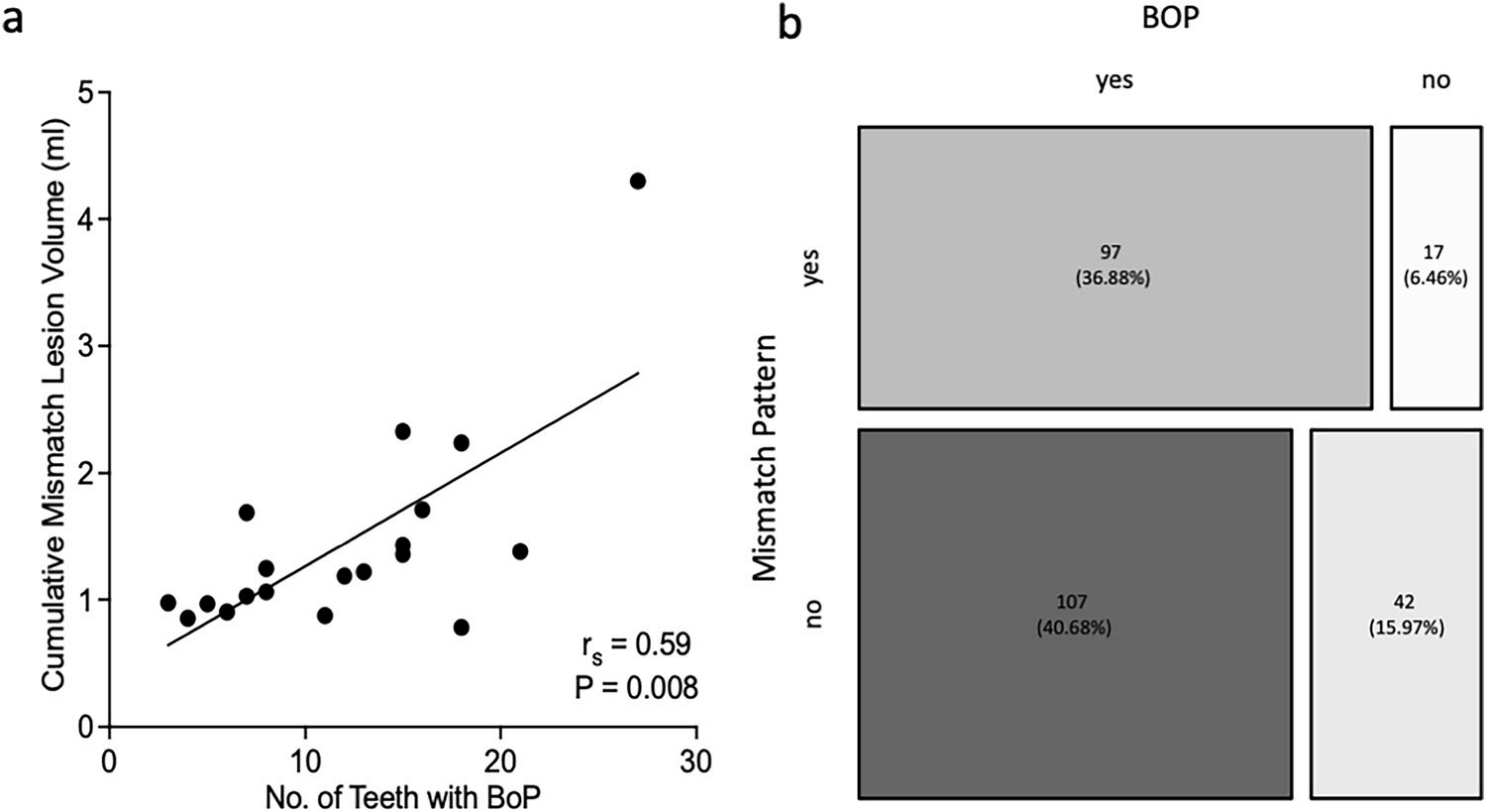
Clinical correlations of mismatch lesion pattern. **a** Plot showing the correlation of individual patients totaled mismatch lesion volume and number of periodontitis-affected teeth with bleeding on probing (BOP, *n* = 19 patients). Line indicates two-tailed Spearman correlation (r_s_ = 0.59 [95% CI: 0.17–0.82], *p* = 0.008). **b** Mosaic plot illustrating presence (yes vs. no) of bleeding on probing (BOP) vs. presence (yes vs. no) of the mismatch lesion pattern

The mismatch lesion pattern showed a strong correlation with numbers of BOP lesions (r_s_ = 0.59 [95% CI: 0.17–0.82], *p* = 0.008, Spearman’s correlation, Fig. 3a) and the individual patient BOP percentage (r_s_ = 0.59 [95% CI: 0.17–0.83], *p* < 0.008, Spearman’s correlation). In multivariate regression models (Table 2), the associations between T2W lesion excess and BOP lesions or the BOP% remained significant after adjustment for history of smoking (overall regression *R*^2^ = 0.71, F (3,15) = 11.96, *p* < 0.001 and *R*^2^ = 0.63, F (3,15) = 8.37, *p* = 0.002, respectively, Table 2). Further, there was an association of mismatch lesions (yes vs. no) with positive BOP (85.8% vs. 70.9%, *p* = 0.005, Fisher’s exact test). Yet a large proportion of BOP-positive lesions did not exhibit the mismatch lesion pattern (Fig. 3b). This would translate into a sensitivity of 45.7% and a specificity of 71.2% and a respective positive predictive value of 85.1% and a negative predictive value of 28.2% for the T2W mismatch marker and BOP.

**Table 2:** Multivariate linear regression analysis of cumulative T2W/T1WC mismatch lesion volume and bleeding on probing

Individual patient numbers of bleeding on probing lesions			
	β	95% CI	*p*-value
Cumulative T2W/T1WC mismatch lesion volume (mL)	63.32	39.56–87.07	< 0.001
History of smoking (yes)	21.55	2.66–40.44	0.028
Age (years)	−0.41	−1.17 to 0.36	0.275

Individual patient bleeding on probing percentage			
	β	95% CI	*p*-value
Cumulative T2W/T1WC mismatch lesion volume (mL)	16.24	8.40–24.09	< 0.001
History of smoking (yes)	3.91	−2.33 to 10.14	0.201
Age (years)	−0.21	−0.46 to 0.041	0.094

## Discussion

Our study demonstrates that dMRI is a reliable tool for assessing volumetric infrabony and interradicular bone lesions in severe periodontitis, showing strong volumetric correlations with CBCT. However, dMRI consistently overestimated lesion size compared to CBCT, with median errors of 37% for T1W + C and 55% for T2W, likely due to its lower precision in defining the margins of osseous bone loss. Despite this limitation, dMRI offers a key advantage: it can detect inflammatory changes in perilesional bone tissue, which are not delineated on x-ray-based imaging. This capability may facilitate earlier therapeutic interventions by identifying inflammation before bone loss becomes evident.

To provide a frame of reference, the observed volumetric overestimation bias of 37–55% corresponds to median linear dimension errors of approximately 11–16% (assuming uniform scaling and applying the two-thirds power rule). This places T1W + C delineated lesions within the range reported in prior studies, where dMRI demonstrated excellent diagnostic performance for both horizontal and vertical bone loss, with reported sensitivity/specificity values of 98%/100% and 99%/99%, respectively, compared to CBCT [12]. Nonetheless, our findings also highlight that T2-weighted imaging appears less suited for precise quantification of bone loss volume. Further research is needed to determine whether this limitation can be mitigated through optimized thresholding or segmentation protocols. Despite this, dMRI offers the key advantage of the detection of inflammatory changes in perilesional bone tissue. This study introduces two novel patterns of periodontal lesions using contrast-enhanced T1-weighted and T2-weighted imaging:

1. In the “mismatch” pattern, T2W MRI signal abnormalities extended well beyond the bone loss boundaries identified by CBCT and exceeded the corresponding changes seen in contrast-enhanced T1-weighted imaging (T2W » T1W + C). These findings are consistent with previous studies indicating that T2W-dMRI is more sensitive in detecting periodontal changes compared to panoramic radiography [14–16]. This pattern demonstrated a strong association with clinical disease activity. However, not all mismatch bone lesions were linked to bleeding on probing (BOP), a common clinical marker of inflammation in periodontal tissues [18]. This suggests that these lesions may serve as an additional biomarker for inflammation, distinct from probing pocket findings.
2. In the “match” pattern (T2W ≈ T1W + C lesions), dMRI lesion volumes closely corresponded to the bone loss defined by CBCT, with a median error of 18.9% for T1W + C imaging. This in line with previous studies that have demonstrated a strong agreement between contrast-enhanced T1-weighted imaging and CBCT in evaluating both horizontal and vertical lesion extension in maxillary molars with furcation involvement [12].

Assessment of periodontal disease activity, treatment response, and clinical decision-making is currently based on clinical parameters such as CAL, PD and BOP, alongside radiographic evaluations. While the combination of clinical and radiographic evidence provides a comprehensive periodontal assessment, important diagnostic limitations remain, especially with regard to disease progression. CAL and PD, together with radiographic imaging, are well-suited to assess cumulative tissue destruction, but lack the capacity to capture momentary disease activity. Conversely, BOP is considered an indicator of active inflammation, yet it provides only dichotomous information, does not allow for grading of severity and is spatially limited to the probing site. Moreover, the positive predictive value of repetitively positive BOP for progressive CAL is only 30%, highlighting the clinical need for better biomarkers to predict disease progression [22]. Multimodal dMRI holds promise in addressing these diagnostic gaps with volumetric data that reflect both cumulative tissue injury and acute, site-specific inflammatory changes. Mismatch lesions were also identified at sites that tested negative for BOP, indicating the potential existence of a subgroup of lesions that may not be recognized as active through clinical examination alone.

With further validation, dMRI could significantly improve clinical decision-making by distinguishing between active progressive versus stable lesions. This differentiation is clinically meaningful, as exclusive reliance on clinical and radiographic parameters has been associated with both overtreatment and undertreatment. The integration of additional biomarkers, including imaging-derived metrics, may therefore be critical for accurately differentiating between stable and progressive forms of periodontitis [23]. Beyond its prognostic value, non-ionizing 3D imaging also offers advantages in surgical and therapeutic planning by providing detailed visualization of defect morphology, depth, and width, aiding in more precise treatment strategies [24]. Supporting this, longitudinal studies have shown that effective non-surgical interventions (evidenced by reduced average probing depths) correlate with a subsequent decrease in periodontal bone marrow edema, as detected through T2-weighted imaging [14–16].

This study has several methodological strengths. In addition to a prospective clinical evaluation, a dedicated dMRI examination protocol was implemented, achieving high spatial resolution (0.6–0.7 mm) with a short scan time of less than 15 min. Furthermore, sequence optimization for metal artifact suppression significantly improved image quality, resulting in the exclusion of only 4.6% of teeth due to metal artifacts, compared to approximately 15% in previous studies [14, 16].

Most notably, this in vivo study is the first to directly compare dMRI findings with CBCT, the clinical 3D reference standard, providing a comprehensive assessment of dMRI’s diagnostic reliability in periodontitis.

The main limitation of our study is the lack of longitudinal data. This limits our ability to determine whether the observed signal abnormalities can serve as early indicators of progressive bone degeneration over time. Additionally, the response of these periodontal signal abnormalities to treatment remains unclear. While Schwarting et al demonstrated that T2W periodontal lesions regress following successful treatment, as indicated by improved probing depths, no longitudinal CBCT data on bony structural changes are available. Repeated CBCT assessments, in the authors’ opinion, would not align with ethical principles in medical research due to radiation exposure.

Another limitation is the relatively small patient cohort, which consists exclusively of individuals with severe periodontitis. This restricts the generalizability of our findings to a broader population. Furthermore, the current availability and cost of dMRI compared to CBCT limit its widespread clinical application. However, anticipated advancements in specialized dental MRI technology are expected to make this modality more accessible in the future [25].

## Conclusion

Reliability in assessing volumetric infrabony and interradicular bone loss in periodontitis using multimodal dMRI is good. A substantial volumetric bone lesion overestimation by dMRI compared to bone loss seen in CBCT was noted, with T1W + C showing a lower overestimation (median lesion overestimation of 37%) than T2W (55%). However, dMRI excels in detecting periodontal disease extent beyond bone loss, offering potential to identify tissue at high risk for degradation. We identified a novel “mismatch lesion pattern” linked to, but distinct from, clinical signs of active inflammation. Longitudinal studies are needed to determine if these patterns correlate with disease progression and therapeutic outcomes.

## Data Availability

The raw imaging and clinical datasets analyzed during the current study are not publicly available due to data privacy laws, but datasets generated are available from the corresponding author upon reasonable request.

## Abbreviations

BOP: Bleeding on probing
CAL: Clinical attachment levels
CBCT: Cone-beam computed tomography
dMRI: Dental MRI
PD: Probing pocket depths
T1W + C: Contrast-enhanced-T1-weighting
T2W: T2-weighting
VOIs: Volumes of interest
